# Code2Inv: A Deep Learning Framework for Program Verification

**DOI:** 10.1007/978-3-030-53291-8_9

**Published:** 2020-06-16

**Authors:** Xujie Si, Aaditya Naik, Hanjun Dai, Mayur Naik, Le Song

**Affiliations:** 8 Microsoft Research Lab, Redmond, WA USA; 9 University of Southern California, Los Angeles, CA USA; 10 University of Pennsylvania, Philadelphia, USA; 11 Google Brain, Mountain View, USA; 12 Georgia Institute of Technology, Atlanta, USA

## Abstract

We propose a general end-to-end deep learning framework Code2Inv, which takes a verification task and a proof checker as input, and automatically learns a valid proof for the verification task by interacting with the given checker. Code2Inv is parameterized with an embedding module and a grammar: the former encodes the verification task into numeric vectors while the latter describes the format of solutions Code2Inv should produce. We demonstrate the flexibility of Code2Inv by means of two small-scale yet expressive instances: a loop invariant synthesizer for C programs, and a Constrained Horn Clause (CHC) solver.

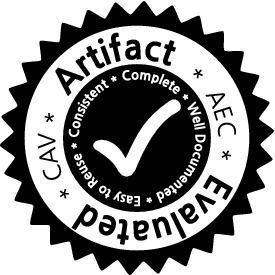



## Introduction

A central challenge in automating program verification lies in effective proof search. Counterexample-guided Inductive Synthesis (CEGIS) [3, 4, 17, 31, 32] has emerged as a promising paradigm for solving this problem. In this paradigm, a *generator* proposes a candidate solution, and a *checker* determines whether the solution is correct or not; in the latter case, the checker provides a counterexample to the generator, and the process repeats.

Finding loop invariants is arguably the most crucial part of proof search in program verification. Recent works [2, 9, 10, 26, 29, 38] have instantiated the CEGIS paradigm for synthesizing loop invariants. Since *checking* loop invariants is a relatively standard process, these works target *generating* loop invariants using various approaches, such as stochastic sampling [29], syntax-guided enumeration [2, 26], and decision trees with templates [9, 10] or linear classifiers [38]. Despite having greatly advanced the state-of-the-art in program verification, however, there remains significant room for improvement in practice.

We set out to build a CEGIS-based program verification framework and identified five key objectives that it must address to be useful:

- The proof search should automatically evolve according to a given verification task as opposed to using exhaustive enumeration or a fixed set of search heuristics common in existing approaches.
- The framework should be able to transfer knowledge across programs, that is, past runs should boost performance on similar programs in the future, which is especially relevant for CI/CD settings [15, 20, 25].
- The framework should be able to adapt to generate different kinds of invariants (e.g. non-linear or with quantifiers) beyond linear invariants predominantly targeted by existing approaches.
- The framework should be extensible to a new domain (e.g. constraint solving-based) by simply switching the underlying checker.
- The generated invariants should be natural, e.g. avoid overfitting due to human-induced biases in the proof search heuristic or invariant structure commonly imposed through templates.

We present Code2Inv, an end-to-end deep learning framework which aims to realize the above objectives. Code2Inv has two key differences compared to existing CEGIS-based approaches. First, instead of simply focusing on counterexamples but ignoring program structure, Code2Inv learns a neural representation of program structure by leveraging graph neural networks [8, 11, 19, 28], which enable to capture structural information and thereby generalize to different but structurally similar programs. Secondly, Code2Inv reduces loop invariant generation into a deep reinforcement learning problem [22, 34]. No search heuristics or training labels are needed from human experts; instead, a neural policy for loop invariant generation can be automatically learned by interacting with the given proof checker on the fly. The learnable neural policy generates a loop invariant by taking a sequence of actions, which can be flexibly controlled by a grammar that defines the structure of loop invariants. This decoupling of the action definition from policy learning enables Code2Inv to adapt to different loop invariants or other reasoning tasks in a new domain with almost no changes except for adjusting the grammar or the underlying checker.

We summarize our contributions as follows:

- We present a framework for program verification, Code2Inv, which leverages deep learning and reinforcement learning through the use of graph neural network, tree-structured long short-term memory network, attention mechanism, and policy gradient.
- We show two small-scale yet expressive instances of Code2Inv: a loop invariant synthesizer for C programs and a Constrained Horn Clause (CHC) solver.
- We evaluate Code2Inv on a suite of 133 C programs from SyGuS [2] by comparing its performance with three state-of-the-art approaches and showing that the learned neural policy can be transferred to similar programs.
- We perform two case studies showing the flexibility of Code2Inv on different classes of loop invariants. We also perform a case study on the naturalness of the loop invariants generated by various approaches.

## Background

In this section, we introduce artificial neural network concepts used by Code2Inv. A multilayer perceptron (MLP) is a basic neural network model which can approximate an arbitrary continuous function $\mathbf {y} = f^*(\mathbf {x})$, where $\mathbf {x}$ and $\mathbf {y}$ are numeric vectors. An MLP defines a mapping $\mathbf {y} = f(\mathbf {x};\mathbf {\theta })$, where $\mathbf {\theta }$ denotes weights of connections, which are usually trained using gradient descent methods.

Recurrent neural networks (RNNs) approximate the mapping from a sequence of inputs $\mathbf {x}^{(1)},...,\mathbf {x}^{(t)}$ to either a single output $\mathbf {y}$ or a sequence of outputs $\mathbf {y}^{(1)},...,\mathbf {y}^{(t)}$. An RNN defines a mapping $\mathbf {h}^{(t)} = f(\mathbf {h}^{(t-1)}, \mathbf {x}^{(t)};\mathbf {\theta })$, where $\mathbf {h}^{(t)}$ is the hidden state, from which the final output $\mathbf {y}^{(t)}$ can be computed (e.g. by a non-linear transformation or an MLP). A common RNN model is the long short-term memory network (LSTM) [16] which is used to learn long-term dependencies. Two common variants of LSTM are gated recurrent units (GRUs) [7] and tree-structured LSTM (Tree-LSTM) [35]. The former simplifies the LSTM for efficiency while the latter extends the modeling ability to tree structures.

In many domains, graphs are used to represent data with rich structure, such as programs, molecules, social networks, and knowledge bases. Graph neural networks (GNNs) [1, 8, 11, 19, 36] are commonly used to learn over graph-structured data. A GNN learns an embedding (i.e. real-valued vector) for each node of the given graph using a recursive neighborhood aggregation (or neural message passing) procedure. After training, a node embedding captures the structural information within the node’s *K*-hop neighborhood, where *K* is a hyper-parameter. A simple aggregation of all node embeddings or pooling [37] according to the graph structure summarizes the entire graph into an embedding. GNNs are parametrized with other models such as MLPs, which are the learnable non-linear transformations used in message passing, and GRUs, which are used to update the node embedding.

Lastly, the generalization ability of neural networks can be improved by an external memory [12, 13, 33] which can be accessed using a differentiable *attention mechanism* [5]. Given a set of neural embeddings, which form the external memory, an attention mechanism assigns a likelihood to each embedding, under a given neural context. These likelihoods guide the selection of decisions that are represented by the chosen embeddings.

## Framework

We first describe the general framework, Code2Inv, and then illustrate two instances, namely, a loop invariant synthesizer for C programs and a CHC solver.

**Fig. 1.**
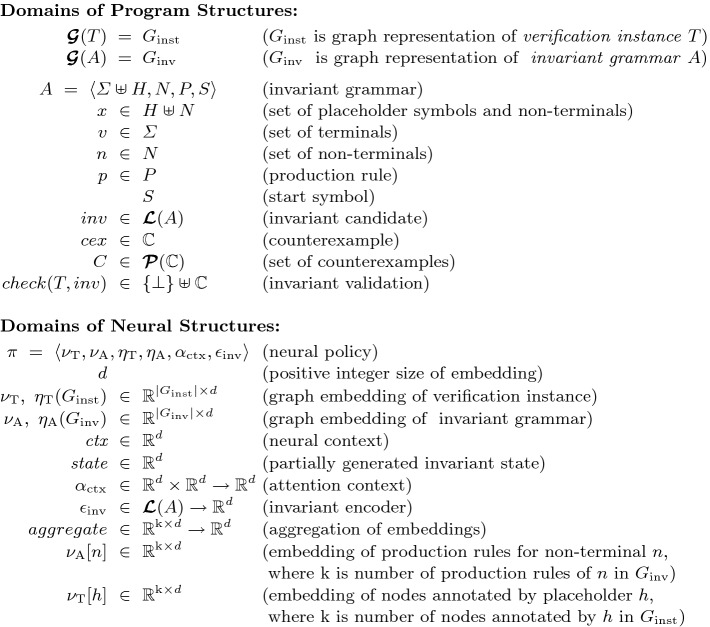
Semantic domains. $\varvec{\mathcal {L}}(A)$ denotes the set of all sentential forms of $A$.


Figure 1 defines the domains of program structures and neural structures used in Code2Inv. The framework is parameterized by graph constructors $\varvec{\mathcal {G}}$ that produce graph representations of verification instance $T$ and invariant grammar $A$, denoted $G_{\text {inst}}$ and $G_{\text {inv}}$, respectively. The invariant grammar uses placeholder symbols *H*, which represent *abstract* values of entities such as variables, constants, and operators, and will be replaced by *concrete* values from the verification instance during invariant generation. The framework requires a black-box function *check* that takes a verification instance $T$ and a candidate invariant *inv*, and returns success (denoted $\bot$) or a counterexample *cex*.

The key component of the framework is a neural policy $$\pi $$ which comprises four neural networks. Two graph neural networks, $$\eta _{\text {T}}$$ and $$\eta _{\text {A}}$$, are used to compute neural embeddings, $$\nu _{\text {T}}$$ and $$ \nu _{\text {A}}$$, for graph representations $$G_{\text {inst}}$$ and $$G_{\text {inv}}$$, respectively. The neural network $$\alpha _{\text {ctx}}$$, implemented as a GRU, maintains the attention context $$ctx $$ which controls the selection of the production rule to apply or the concrete value to replace a placeholder symbol at each step of invariant generation. The neural network $$\epsilon _{\text {inv}}$$, implemented as a Tree-LSTM, encodes the partially generated invariant into a numeric vector denoted $$state $$, which captures the state of the generation that is used to update the attention context $$ctx $$.

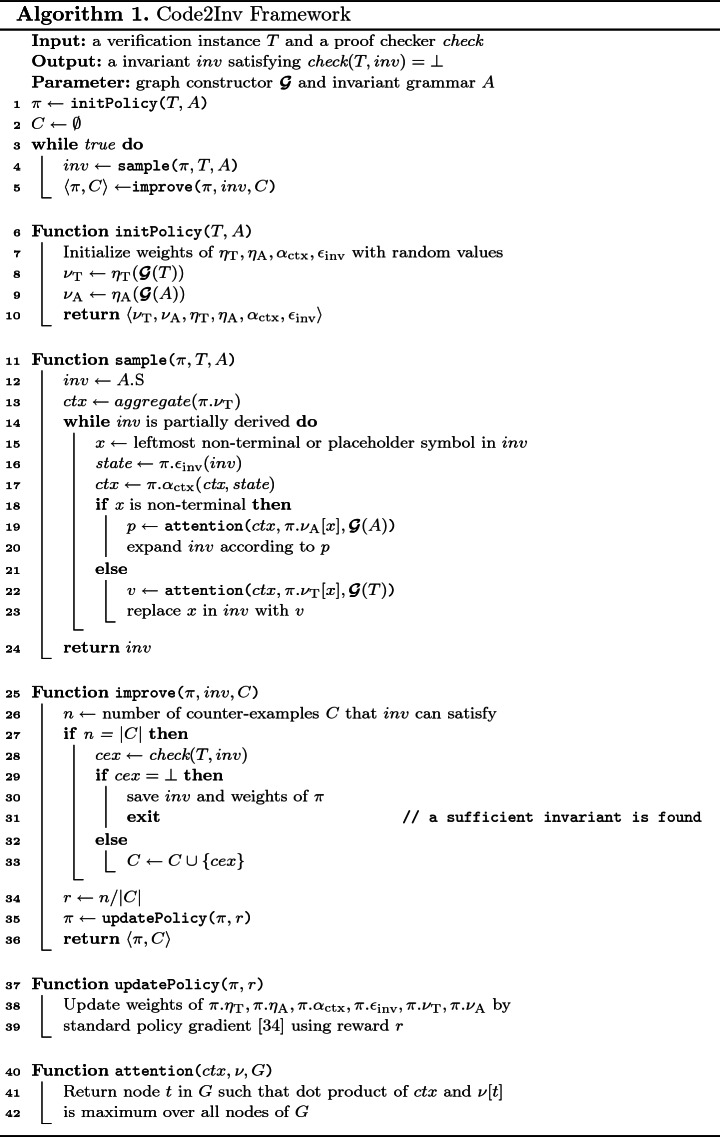


Algorithm 1 depicts the main algorithm underlying Code2Inv. It takes a verification instance and a proof checker as input and produces an invariant that suffices to verify the given instance^1^. At a high level, Code2Inv learns a neural policy, in lines 1–5. The algorithm first initializes the neural policy and the set of counterexamples (line 1–2). The algorithm then iteratively samples a candidate invariant (line 4) and improves the policy using a reward for the new candidate based on the accumulated counterexamples (line 5). We next elucidate upon the initialization, policy sampling, and policy improvement procedures.

**Initialization.** The initPolicy procedure (line 6–10) initializes the neural policy. All four neural networks are initialized with random weights (line 7), and graph embeddings $\nu _{\text {T}}, \nu _{\text {A}}$ for verification task $T$ and invariant grammar $A$ are computed by applying corresponding graph neural networks $\eta _{\text {T}}, \eta _{\text {A}}$ to their graph representations $\varvec{\mathcal {G}}(T), \varvec{\mathcal {G}}(A)$ respectively. Alternatively, the neural networks can be initialized with pre-trained weights, which can boost overall performance.

**Neural Policy Sampling.** The sample procedure (lines 11–24) generates a candidate invariant by executing the current neural policy. The candidate is first initialized to the start symbol of the given grammar (line 12), and then updated iteratively (lines 14–23) until it is complete (i.e. there are no non-terminals). Specifically, the candidate is updated by either expanding its leftmost non-terminal according to one of its production rules (lines 19–20) or by replacing its leftmost placeholder symbol with some concrete value from the verification instance (lines 22–23). The selection of a production rule or concrete value is done through an *attention mechanism*, which picks the most likely one according to the current context and corresponding region of external memory. The neural context is initialized to the aggregation of embeddings of the given verification instance (line 13), and then maintained by $\alpha _{\text {ctx}}$ (line 17) which, at each step, incorporates the neural state of the partially generated candidate invariant (line 16), where the neural state is encoded by $\epsilon _{\text {inv}}$.

**Neural Policy Improvement.** The improve procedure (lines 25–36) improves the current policy by means of a *continuous* reward. Simply checking whether the current candidate invariant is sufficient or not yields a discrete reward of 1 (yes) or 0 (no). This reward is too sparse to improve the policy, since most candidate invariants generated are insufficient, thereby almost always yielding a zero reward. Code2Inv addresses this problem by accumulating counterexamples provided by the checker. Whenever a new candidate invariant is generated, Code2Inv tests the number of counterexamples it can satisfy (line 26), and uses the fraction of satisfied counterexamples as the reward (line 34). If all counterexamples are satisfied, Code2Inv queries the checker to validate the candidate (line 28). If the candidate is accepted by the checker, then a sufficient invariant was found, and the learned weights of the neural networks are saved for speeding up similar verification instances in the future (lines 29–31). Otherwise, a new counterexample is accumulated (line 33). Finally, the neural policy (including the neural embeddings) is updated based on the reward.

**Framework Instantiations.** We next show two instantiations of Code2Inv by customizing the graph constructor $\varvec{\mathcal {G}}$. Specifically, we demonstrate two scenarios of graph construction: 1) by carefully exploiting task specific knowledge, and 2) with minimum information of the given task.

**Fig. 2.**
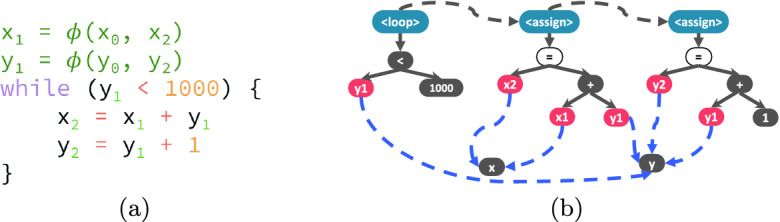
(a) C program snippet in SSA form; (b) its graph representation.

*Instantiation to Synthesize Loop Invariants for C Programs.* An effective graph representation for a C program should reflect its control-flow and data-flow information. We leverage the static single assignment (SSA) transformation for this purpose. Figure 2 illustrates the graph construction process. Given a C program, we first apply SSA transformation as shown in Fig. 2a, from which a graph is constructed as shown in Fig. 2b. The graph is essentially abstract syntax trees (ASTs) augmented with control-flow (black dashed) edges and data-flow (blue dashed) edges. Different types of edges will be modeled as different message passing channels used in graph neural networks so that rich structural information can be captured more effectively by the neural embeddings. Furthermore, certain nodes (marked black) are annotated with placeholder symbols and will be used to fill corresponding placeholders during invariant generation. For instance, variables x and y are annotated with VAR, integer values 1000 and 1 are annotated with CONST, and the operator  is annotated with OP.

**Fig. 3.**
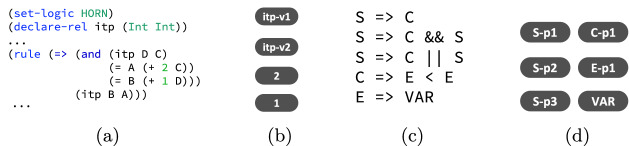
(a) CHC instance snippet; (b) node representation for the CHC example; (c) example of invariant grammar; (d) node representation for the grammar.

*Instantiation to Solve Constrained Horn Clauses (CHC).* CHC are a uniform way to represent recursive, inter-procedural, and multi-threaded programs, and serve as a suitable basis for automatic program verification [6] and refinement type inference [21]. Solving a CHC instance involves determining unknown predicates that satisfy a set of logical constraints. Figure 3a shows a simple example of a CHC instance where *itp* is the unknown predicate. It is easy to see that *itp* in fact represents an invariant of a loop. Thus, CHC solving can be viewed as a generalization of finding loop invariants [6].

Unlike C programs, which have explicit control-flow and data-flow information, a CHC instance is a set of *un-ordered* Horn rules. The graph construction for Horn rules is not as obvious as for C programs. Therefore, instead of deliberately constructing a graph that incorporates detailed domain-specific information, we use a *node representation*, which is a degenerate case of graph representation and requires only necessary nodes but no edges. Figure 3b shows the node representation for the CHC example from Fig. 3a. The top two nodes are derived from the signature of unknown predicate *itp* and represent the first and the second arguments of *itp*. The bottom two nodes are constants extracted from the Horn rule. We empirically show that node representation works reasonably well. The downside of node representation is that no structural information is captured by the neural embeddings which in turn prevents the learned neural policy from generalizing to other structurally similar instances.

*Embedding Invariant Grammar.* Lastly, both instantiations must define the embedding of the invariant grammar. The grammar can be arbitrarily defined, and similar to CHCs, there is no obvious information such as control- or data-flow to leverage. Thus, we use node representation for the invariant grammar as well. Figure 3c and Fig. 3d shows an example of invariant grammar and its node representation, respectively. Each node in the graph represents either a terminal or a production rule for a non-terminal. Note that this representation does not prevent the neural policy from generalizing to similar instances as long as they share the same invariant grammar. This is feasible because the invariant grammar does not contain instance specific details, which are abstracted away by placeholder symbols like VAR, CONST, and OP.

## Evaluation

We first discuss the implementation, particularly the improvement over our previous prototype [30], and then evaluate our framework in a number of aspects, such as performance, transferability, flexibility, and naturalness.

**Implementation.** Code2Inv^2^ consists of a frontend, which converts an instance into a graph, and a backend, which maintains all neural components (i.e. neural embeddings and policy) and interacts with a checker. Our previous prototype has a very limited frontend based on CIL [24] and no notion of invariant grammar in the backend. We made significant improvements in both the frontend and the backend. We re-implemented the frontend for C programs based on Clang and implemented a new frontend for CHCs. We also re-implemented the backend to accept a configurable invariant grammar. Furthermore, we developed a standard graph format, which decouples the frontend and backend, and a clean interface between the backend and the checker. No changes are needed in the backend to support new instantiations.

**Evaluation Setup.** We evaluate both instantiations of Code2Inv by comparing each instantiation with corresponding state-of-the-art solvers. For the task of synthesizing loop invariants for C programs, we use the same suite of benchmarks from our previous work [30], which consists of 133 C programs from SyGuS [2]. We compare Code2Inv with our previous specialized prototype and three other state-of-the-art verification tools: C2I [29], LoopInvGen [26] and ICE-DT [10]. For the CHC solving task, we collect 120 CHC instances using SeaHorn [14] to reduce the C benchmark programs into CHCs.^3^ We compare Code2Inv with two state-of-the-art CHC solvers: Spacer [18], which is the default fixedpoint engine of Z3, and LinearyArbitrary [38]. We run all solvers on a single 2.4 GHz AMD CPU core up to 12 h and using up to 4 GB memory. Unless specified otherwise, Code2Inv is always initialized randomly, that is, untrained.

**Fig. 4.**
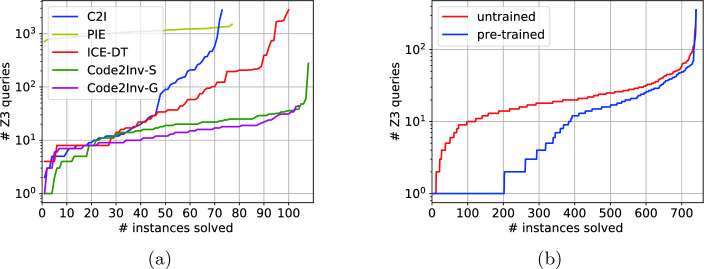
(a) Comparison of Code2Inv with state-of-the-art solvers; (b) comparison between untrained model and pre-trained model.

**Performance.** Given that both the hardware and the software environments could affect the absolute running time and that all solvers for loop invariant generation for C programs rely on the same underlying SMT engine, Z3 [23], we compare the performance in terms of number of Z3 queries. We note that this is an imperfect metric but a relatively objective one that also highlights salient features of Code2Inv. Figure 4a shows the plot of verification cost (i.e. number of Z3 queries) by each solver and the number of C programs successfully verified within the corresponding cost. Code2Inv significantly outperforms other state-of-the-art solvers in terms of verification cost and the general framework Code2Inv-G achieves performance comparable to (slightly better than) the previous specialized prototype Code2Inv-S.

**Transferability**. Another hallmark of Code2Inv is that, along with the desired loop invariant, it also learns a neural policy. To evaluate the performance benefits of the learned policy, we randomly perturb the C benchmark programs by various edits (e.g. renaming existing variables and injecting new variables and statements). For each program, we obtain 100 variants, and use 90 for training and 10 for testing. Figure 4b shows the performance difference between the untrained model (i.e. initialized with random weights) and the pre-trained model (i.e. initialized with pre-trained weights). Our results indicate that the learned neural policy can be transferred to accelerate the search for loop invariants for similar programs. This is especially useful in the CI/CD setting [25] where programs evolve incrementally and quick turnaround time is indispensable.

**Flexibility.** Code2Inv can be instantiated or extended in a very flexible manner. For one instance, with a simple frontend (e.g. node representation as discussed above), Code2Inv can be customized as a CHC solver. Our evaluation shows that, without any prior knowledge about Horn rules, Code2Inv can solve 94 (out of 120) CHC instances. Although it is not on a par with state-of-the-art CHC solvers Spacer and LinearArbitrary, which solve 112 and 118 instances, respectively, Code2Inv provides new insights for solving CHCs and could be further improved by better embeddings and reward design.

As another example, by simply adjusting the invariant grammar, Code2Inv is immediately ready for solving CHC tasks involving *non-linear* arithmetic. Our case study shows that Code2Inv successfully solves 5 (out of 7) non-linear instances we created^4^, while both Spacer and LinearArbitrary failed to solve any of them. Tasks involving non-linear arithmetic are particularly challenging because the underlying checker is more likely to get stuck, and no feedback (e.g. counterexample) can be provided, which is critical for existing solvers like Spacer and LinearArbitrary to make progress. This highlights another strength of Code2Inv—even if the checker gets stuck, the learning process can still continue by simply assigning zero or negative reward.

**Fig. 5.**
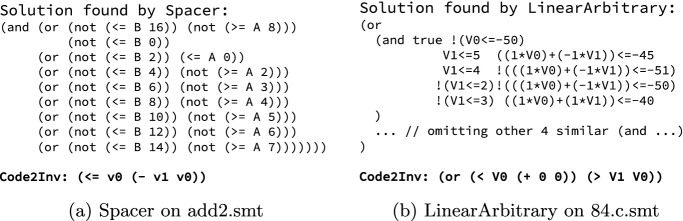
Comparison of solution naturalness.

**Naturalness.** Our final case study concerns the naturalness of solutions. As illustrated in Fig. 5, solutions discovered by Code2Inv tend to be more natural, whereas Spacer and LinearArbitrary tend to find solutions that unnecessarily depend on constants from the given verification instance. Such *overfitted* solutions may become invalid when these constants change. Note that expressions such as (+ 0 0) in Code2Inv’s solutions can be eliminated by post-processing simplification akin to peephole optimization in compilers. Alternatively, the reward mechanism in Code2Inv could incorporate a regularizer on the naturalness.

**Limitations.** Code2Inv does not support finding loop invariants for programs with multiple loops, function calls, or recursion. Code2Inv generally runs slower compared to other contemporary approaches. Specifically, 90% of the solved C instances took 2 h or less, and the rest could take up to 12 hours to solve. This could be improved upon by leveraging GPUs, developing more efficient training algorithms, or leveraging templates [27].

## Conclusion

We presented a framework Code2Inv which automatically learns invariants (or more generally unknown predicates) by interacting with a proof checker. Code2Inv is a general and learnable tool for solving many different verification tasks and can be flexibly configured with a grammar and a graph constructor. We compared its performance with state-of-the-art solvers for both C programs and CHC formulae, and showed that it can adapt to different types of inputs with minor changes. We also showed, by simply varying the input grammar, how it can tackle non-linear invariant problems which other solvers are not equipped to work with, while still giving results that are relatively natural to read.

## Footnotes

1 Fuzzers may be applied first so that the confidence of existence of a proof is high.

2 Our artifacts are available on GitHub: https://github.com/PL-ML/code2inv.

3 SeaHorn produces empty Horn rules on 13 (out of 133) C programs due to optimizations during VC generation that result in proving the assertions of interest.

4 The non-linear instances we created are available in the artifact.
